# Straw Mulching Reduces the Harmful Effects of Extreme Hydrological and Temperature Conditions in Citrus Orchards

**DOI:** 10.1371/journal.pone.0087094

**Published:** 2014-01-28

**Authors:** Yi Liu, Jing Wang, Dongbi Liu, Zhiguo Li, Guoshi Zhang, Yong Tao, Juan Xie, Junfeng Pan, Fang Chen

**Affiliations:** 1 Laboratory of Aquatic Botany and Watershed Ecology, Wuhan Botanical Garden, Chinese Academy of Sciences China, Wuhan, China; 2 Institute of Plant Protection and Soil Fertilizer, Hubei Academy of Agricultural Sciences, Wuhan, China; 3 China Program, International Plant Nutrition Institute (IPNI), Wuhan, China; Wuhan Botanical Garden, Chinese Academy of Sciences China, China

## Abstract

Extreme weather conditions with negative impacts can strongly affect agricultural production. In the Danjiangkou reservoir area, citrus yields were greatly influenced by cold weather conditions and drought stress in 2011. Soil straw mulching (SM) practices have a major effect on soil water and thermal regimes. A two-year field experiment was conducted to evaluate whether the SM practices can help achieve favorable citrus fruit yields. Results showed that the annual total runoff was significantly (P<0.05) reduced with SM as compared to the control (CK). Correspondingly, mean soil water storage in the top 100 cm of the soil profile was increased in the SM as compared to the CK treatment. However, this result was significant only in the dry season (Jan to Mar), and not in the wet season (Jul to Sep) for both years. Interestingly, the SM treatment did not significantly increase citrus fruit yield in 2010 but did so in 2011, when the citrus crop was completely destroyed (zero fruit yield) in the CK treatment plot due to extremely low temperatures during the citrus overwintering stage. The mulch probably acted as an insulator, resulting in smaller fluctuations in soil temperature in the SM than in the CK treatment. The results suggested that the small effects on soil water and temperature changes created by surface mulch had limited impact on citrus fruit yield in a normal year (e.g., in 2010). However, SM practices can positively impact citrus fruit yield in extreme weather conditions.

## Introduction

One of the major challenges of climate change to agriculture and food security involves ever increasing extreme weather events world widely, such as droughts, heat waves, excessive cold, heavy and prolonged rainfalls, hailstorms, and so on [1]. They usually cause negative effects and sometimes fatal damages to crops, physiologically and/or physically [2]. However, the stresses could be relieved and neutralized by certain positive effects from field microclimate, beneficial soil water and thermal conditions under some farmland management practices [3]. Hence, there is an increasing need for understanding the response of soil water and temperature dynamics to changes in extreme weather conditions [4].

Soil water is considered to be one of the most important factors affecting plant growth and development [5], [6]. Even a small change in soil water storage could greatly affect crop productivity [7]. Soil surface mulching, such as with plastic film [8], crop residue [9], or gravel and sand [10], has a large impact on many of the hydrological and biological processes of soil ecosystems, and the most prominent of these changes is the modification of the soil–plant–atmosphere continuum (SPAC) water cycling. For example, numerous reports indicate that soil straw mulching (SM) favorably influences the soil moisture regime by reducing evaporation from the soil surface [11], [12], improving infiltration [13], and soil water retention [14]. SM has also led to improvements in crop yields in arid and semi-arid environments [5], [12] and economized the use of irrigation water [14]. Thus, a better understanding of the impact of SM practices on soil hydrological processes is becoming critical, especially from the crop production perspective, because of the increasing shortage of water resources worldwide.

Soil temperature controls the rate of crop development, particularly when the meristem is within the soil [15]. Higher soil temperature accelerated the rates of leaf tip appearance and full leaf expansion, enabling the crop to attain maximum green leaf area index more rapidly [10]. SM has been reported to cause either a decrease, an increase or a negligible effect on soil temperature. For instance, SM during over-wintering period can improve soil thermal regime according to several studies [12], [16], [17]. However, Sarkar et al. [18] reported that SM could reduce soil temperature, while effective soil water conservation with SM may result in higher production. While Ghosh et al. [19] argued SM had little or no effect on soil temperature, and that its effects were almost entirely due to increased organic matter. These inconsistent results may depend on multiple factors including soil properties, climate, and species planted [15]. Lower soil temperature under SM has mostly been attributed to the reduced solar energy reaching the soil during hot periods, while increased soil temperature under SM has been attributed to the reduction in outgoing heat radiation from the soil during cold periods.

The Danjiangkou reservoir, established in the 1970s with a drainage area of 95,200 km^2^, is a water source area for China's Middle Route of the South-to-North Water Transfer Project [20]. The staple crops of the region (wheat and rice) are generally grown in the flat land part of the Danjiangkou reservoir area. Citrus is one of the main types of fruit growing on sloping lands, and its high yields, averaging about 40 t hm^−2^, are assumed to be a result of the beneficial thermal effects of the great lakes. After the wheat (or rice) harvest, farmers would generally burn the stalks, but this practice is now prohibited to restore and protect the Danjiangkou reservoir riparian ecosystem. Therefore, farmers have been using wheat (or rice) straw for mulching in citrus orchards. Notably, SM practices can effectively contribute to water conservation and decreased nutrient losses on sloping lands [21]. Although several studies have reported changes in water quality [22] and the role of surface mulching in soil nutrient losses [23], little is known about the impact of SM practices on reducing the harmful effects of extreme weather conditions. In the Danjiangkou reservoir area, citrus yields were strongly affected by cold and drought stress in 2011. The average temperature measured at the Danjiangkuo city meteorological station was 1.5°C during December 2010 to February 2011, which broke the record set in year 2000 and lowered the long-term (10 years) average temperature by 3°C for the corresponding period.

In the present study, we analyzed runoff, soil water content and storage, and seasonal variations in soil temperature under SM and non-mulching or control (CK) treatments. We specifically focused on the role of SM on soil water and temperature dynamics by comparing fruit yield under mulching and no mulching practices. This was done to test the hypothesis that the small effects on soil water conservation and thermoregulation created by surface SM practices can greatly impact citrus fruit yields in extreme weather conditions. The objectives of this study were: (i) to determine how productivity of citrus fruit was affected by soil water and temperature, (ii) to evaluate the influence of SM on the soil water and temperature in sloping citrus orchards in the Danjiangkou Reservoir area.

## Materials and Methods

### Site description

**Figure 1:**
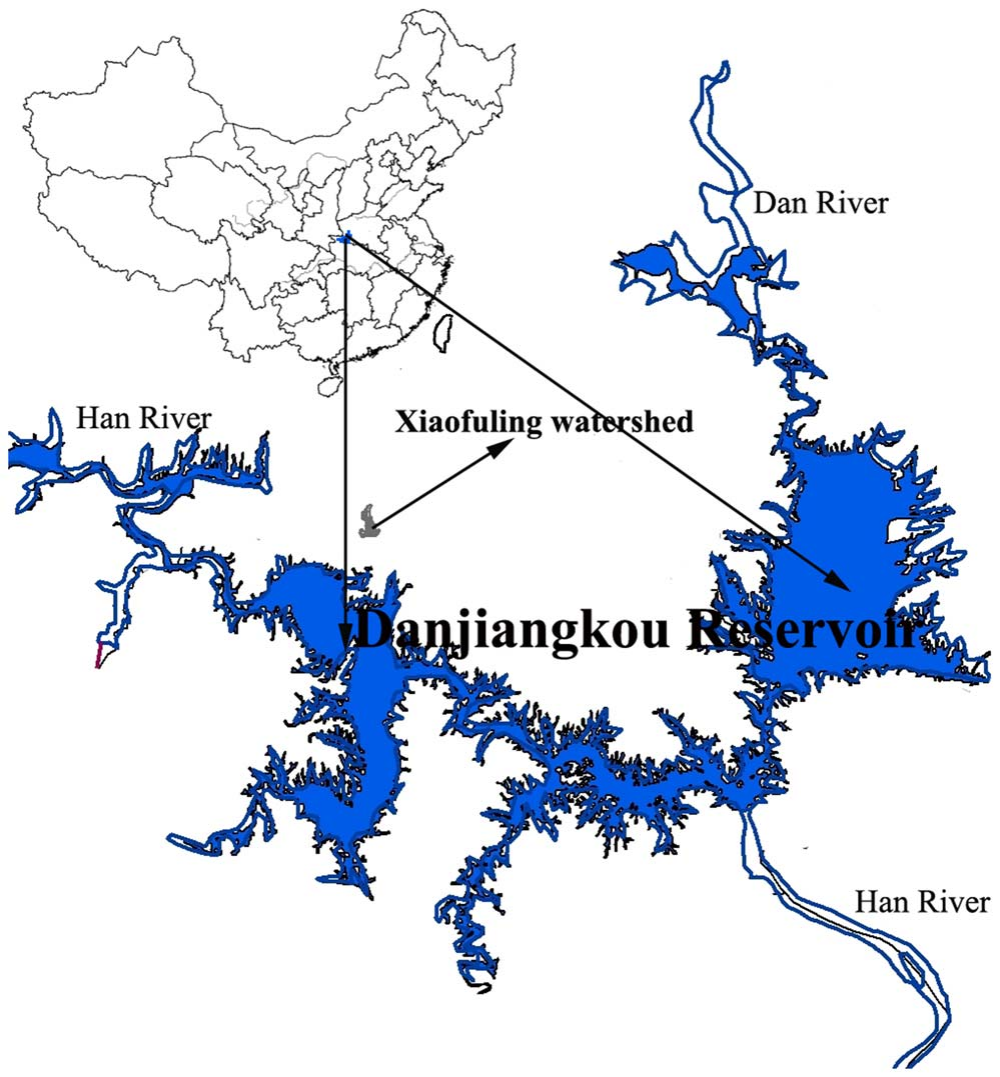
Location of the study area.

This study was conducted in 2010 and 2011 at the Xiaofuling experimental station (32°45′46″N, 111°9′26″E) in the Danjiangkou reservoir area (Fig. 1). The area has a subtropical zone climate with mean annual temperature of 15.7°C, and a monthly average temperature of 27.3°C in July and 4.2°C in January. Mean annual rainfall is approximately 834 mm, 80% of which concentrates between May and October. The study was carried out in a 10-year-old citrus orchard. The experimental site is owned by Wuhan Botanical Garden, Chinese Academy of Sciences China. The field studies did not involve endangered or protected species and no specific permits were required for the described field studies. The site is located about 300 m asl, with an average slope of 15°. The soil at the site is a cinnamon yellow soil as defined by the Chinese soil classification system [24] and textural composition is 14% clay, 23% silt, and 64% sand. At start of the field experiment in 2009, the soil had a pH of 6.5 and a bulk density of 1.45 g cm^−3^. The amounts of organic matter, total nitrogen, available phosphorus, available potassium, and inorganic nitrogen were 9.1 g kg^−1^, 0.88 g kg^−1^, 16.0 mg kg^−1^, 106.3 mg kg^−1^ and 101.8 mg kg^−1^, respectively. These nutrient contents using routine analytical methods [25].

### Experimental treatments and field management

**Figure 2:**
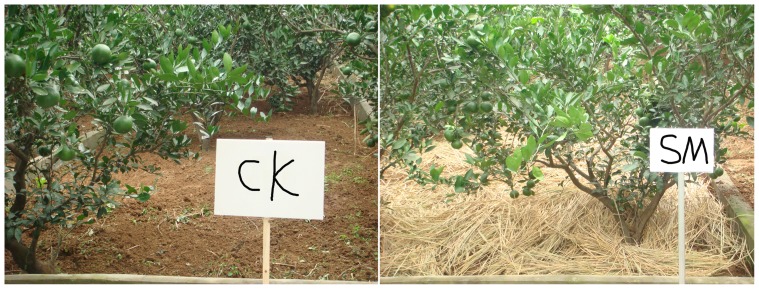
Photographs showing plots under control or without mulching (CK) and straw mulching (SM) treatments.

The experiment was designed with two treatments, CK or without mulching and SM, (Fig. 2) with three replicates for each treatment. All plots, separated by concrete borders, were set up with a tank at the base of each plot to collect the runoff. The size of each experimental plot was 40.5 m^2^ (4.5 m×9 m). The plantation consisted of trained citrus trees at 1.5 m×3 m spacing, with rows perpendicular to the slope. Hence, there are eight trees at each plot. In the plots mulched with straw, rice (or wheat) straw was uniformly applied at a rate of 6,000 kg hm^−2^. Each citrus tree was fertilized in April by hand, with 0.5 kg N (urea), 0.3 kg P_2_O_5_ (superphosphate), and 0.4 kg K_2_O (potassium chloride) in both years (2010 and 2011). Manual weeding was undertaken as required during the citrus growing season.

### Sampling measurements and data calculation

To measure the runoff caused by rainfall, a standard recording rain gauge was sited about 100 m from the experimental plots. Rainfall was calculated at 1-day intervals. Runoff was collected at each plot after each rainfall event, and the depth of water in the runoff collection tank was recorded to calculate runoff volumes. The runoff were determined by the following formula: *R = S*×*h*×10*/Pa,* where *R* is runoff (mm), *S* is collection tank floorage (m^2^), *h* is water depth (cm), *Pa* is plot area (m^2^). The monthly soil water content was determined gravimetrically by oven drying (105°C for 24 h) the core samples that were taken at depth intervals of 20 cm down the 0–100 cm profile in each plot on the 26^th^ (or 27^th^) day of each month (January 2010–December 2011). The soil water storage (*W*) in the profile was considered to be the total storage in all of the sampled layers in the plot, as was calculated using the formula: *W* = *h*×*ρ*×*θ*×1000, where *h* is soil depth (cm), *ρ* is soil bulk density (g cm^−3^), *θ* is soil gravimetric water content (g g^−1^). Soil bulk density was determined from the inner diameter of the core sampler, segment depth, and the oven-dry weight of the core samples at start of the field experiment in 2009 (see Table S1). Air temperature within the canopy (CA, height = 1 m) and soil temperature at 5 cm depth were also recorded at 0.5 h intervals by the StowAway TidbiT temperature recorder (Range: −20° to 70°C in air; −20° to 30°C in water; Accuracy: ±0.4° at 20°C). During the 24-h period, the values were averaged to calculate mean daily temperature.

At the time of commercial harvest, the citrus fruits were harvested gradually when they were ripe. The yield per plot (t hm^−2^) was obtained by weighing the harvested fruit. A random sample of 25 fruits from each plot was collected to determine the average fruit weight. In addition, fruit size was measured by measuring the equatorial diameter with the help of Vernier caliper from each experimental plot. Using a standard juicer, 25 fruits were juiced. The juice was weighed and expressed as a percentage of the total fruit weight.

### Statistical analyses

Statistically significant differences in mean runoff, soil water storage, and soil temperature between the CK and SM treatments were determined utilizing the Wilcoxon signed rank test (WSRT). A P value of <0.05 was considered statistically significant. Statistical analysis for fruit yield and quality was performed using Analysis of Variance (ANOVA). One-way analysis of variance was carried out to determine the differences between the measured parameters for different treatments. Least significant difference (LSD) at P = 0.05 was used to elucidate any significant differences.

## Results

### Rainfall and air temperature

**Figure 3:**
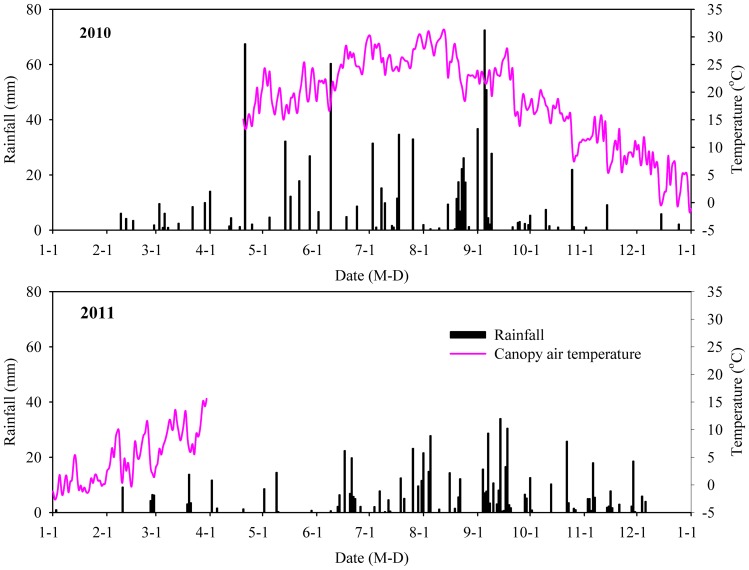
Canopy air temperature and rainfall data for 2010 and 2011 at Xiaofuling experimental station in Danjiangkou reservoir area in Hubei, China. Please note that the record of the air temperatures ended on March 31, 2011 due to the loss of the logger.

The air temperature and rainfall of experimental field are shown in Fig. 3. Field data as well as summary statistics of air temperature and rainfall are provided as Table S2 and S3 in supporting information. The annual precipitation recorded at the experimental site was 849.3 mm in 2010 and 655.5 mm in 2011. 2011 was a dry year, which lowered precipitation by 21.5% compared with the long-term average. Fig. 3 also shows the air temperature within the canopy from April 20, 2010 to March 31, 2011. It should be noted that the record of the air temperatures ended on March 31, 2011 due to the loss of the logger. The daily air temperature varied from −2.5 to 31.2°C, averaging 15.0°C. The average minimum air temperature in the winter of 2010 (i.e., from December 2010 to February 2011) was −2.0°C. There were 66 days with minimum temperature <0°C during the study period, and not only the mean but also the maximum/minimum air temperatures were lower compared to the average of the last 10 years for the corresponding period.

### Runoff

**Figure 4:**
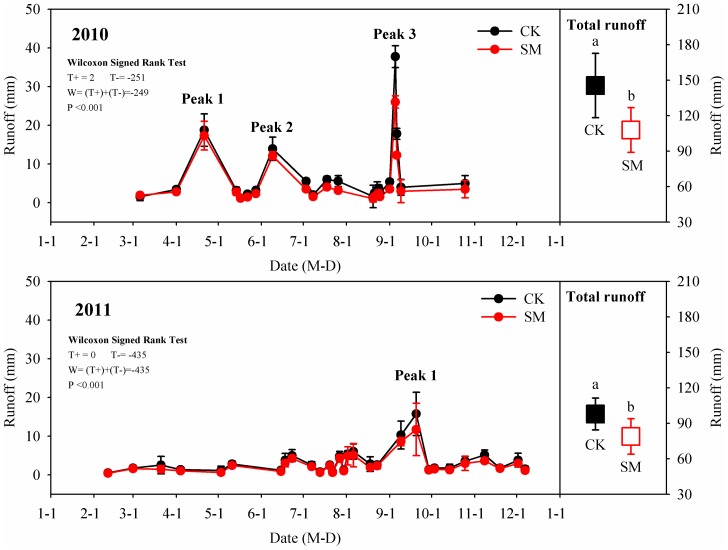
Seasonal variation in and annual total runoff under control (CK) and straw mulching (SM) treatments during 2010 and 2011. Error bars are twice the standard error of the mean (n = 3). Statistically significant differences are given after Wilcoxon signed rank test; Notations a and b indicate statistical significance at P<0.05 between CK and SM.

The surface mean runoff ranged from 1.0 to 37.7 mm in 2010 and from 0.4 to 15.8 mm in 2011 (see Fig. 4 or Table S4), and had three peak values with time (on April 21, June 9, and September 5) in 2010 and one peak value with time (on September 20) in 2011 indicating extreme rainfall events. Three rainstorm events were registered in 2010 as 67.7 mm (April 21, 2010), 60.4 mm (June 9, 2010) and 72.5 mm (September 5, 2010). While one continuous 7 day (From September 13 to 20) rainfall was registered in 2011 as 86.8 mm for accumulative precipitation (see Table S3). Annual total runoff volumes, calculated by adding the readings taken at the sampled points throughout the whole year, were much lower in the SM (107 mm in 2010, 78 mm in 2011) than in the CK (145 mm in 2010, 97 mm in 2011) plots (see Table S4). Lower surface runoff values observed in the SM plots were probably due to good ground coverage and slightly higher water infiltration than in the CK plots.

### Soil water

**Figure 5:**
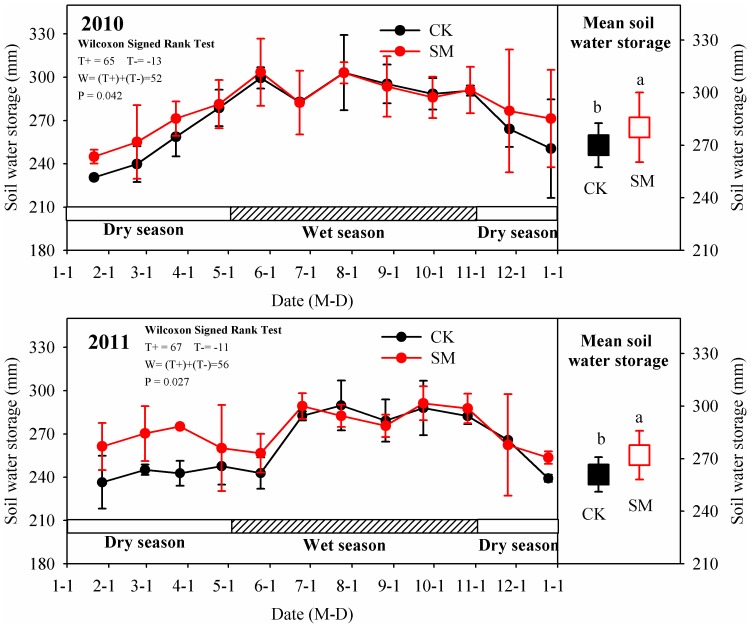
Seasonal variation in and mean soil water storage in the 0–100 cm soil profile under control (CK) and straw mulching (SM) treatments during 2010 and 2011. Error bars are twice the standard error of the mean (n = 3). Statistically significant differences are given after Wilcoxon signed rank test; Notations a and b indicate statistical significance at P<0.05 between CK and SM.

The seasonal variations in water storage in the soil profile (0–100 cm) under CK and SM treatments are shown in Fig. 5. Field data as well as summary statistics of soil water storage are provided as Table S5 in supporting information. Mean soil water storage values, calculated by averaging the readings taken at the sampled points over one year, were much higher in the SM (ranged from 245 to 303 mm in 2010, and 254 to 291 mm in 2011, see Table S5) treatment than in the CK (ranged from 231 to 303 mm in 2010, and 237 to 290 mm in 2011, see Table S5) treatment. Soil water storage exhibited pronounced seasonal variations with minimum values at 231±2 mm during the dry season and maximum values at 303±26 mm during the wet season (Fig. 5). Largest differences in soil water storage between CK and SM occurred during the dry season (from January to March in 2011), when soil under the SM treatment had about 10% (ranged from 5 to 13%) greater water storage than under the CK treatment. During the rainy season in the Danjiangkou reservoir area (July to September), citrus plants take up a great deal of water to maintain their luxuriant growth; the variation in seasonal soil water storage was therefore mainly affected by the amount of precipitation and citrus growth. As a result, no significant difference in soil water storage was observed between CK and SM treatments during the wet season.

**Figure 6:**
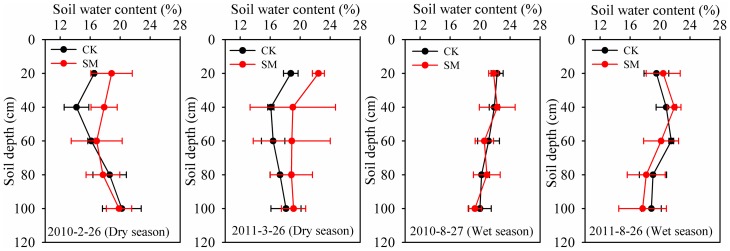
Differences in soil water content down the profile (0–100 cm) in dry (26^th^ February 2010 and 26^th^ March 2010) and wet (27^th^ August 2010 and 26^th^ August 2011) seasons between control (CK) and straw mulching (SM) treatment plots. Error bars are twice the standard error of the mean (n = 3).

The vertical distribution of soil water in a profile under both dry (26^th^ February 2010 and 26^th^ March 2011) and wet (27^th^ August 2010 and 26^th^ August 2011) seasons is shown in Fig. 6. Soil water distribution within the profile results from the combined effects of precipitation amount and movement of soil water. The soil water content was significantly higher in the SM treatment than in the CK treatment in the 0–40 cm soil layer during the dry season, indicating that SM reduced evaporation during the dry period because of the increased surface residue cover and/or the lack of soil disturbance. However, there was no significant difference in the soil water content in all soil layers between the SM and CK treatments during the wet season.

### Soil temperature

**Figure 7:**
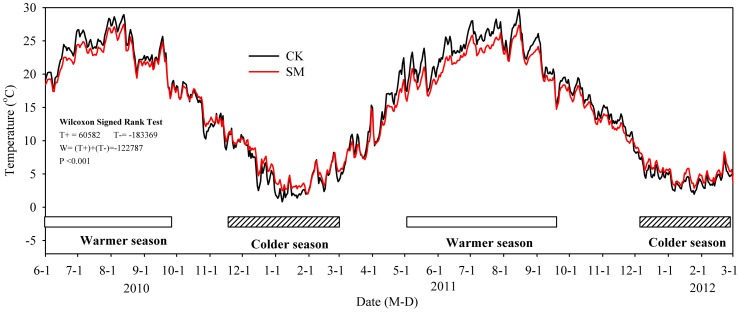
Seasonal variations in soil temperature under control (CK) and straw mulching (SM) treatment plots from June 2010 to March 2012. Statistically significant differences are given after Wilcoxon signed rank test; a P value of <0.05 was considered statistically significant.

The presence of SM altered soil temperature. Fig.7 shows the seasonal variations in soil temperature under CK and SM treatments. Monthly mean soil temperature as well as P values of WSRT are provided as Table 1. Our study demonstrated that soil temperatures under SM plots were higher during the colder seasons and lower during the warmer seasons when compared with the soil temperatures under CK plots. During the warmer period, reductions in soil temperature under the SM treatment were as high as 1.5°C (ranged from 0.3 to 2.9°C) as compared to the CK treatment (see Table S6). Because straw, that covered the soil surface, has a higher albedo and lower thermal conductivity than the bare soil, it helps to reduce the solar energy reaching the soil and, as a result, reduces temperature increases during warm conditions. Conversely, during the colder seasons, the presence of SM on the soil surface insulates the soil from the colder air temperatures. Therefore, heat loss from the soil is somewhat lower and soil temperatures are consequently higher under SM than under CK.

**Table 1:** Monthly mean soil temperature under the control (CK) and straw mulching (SM) treatments from June 2010 to March 2012.

Month-year	Soil temperature (°C)		WSRTP	Month-year	Soil temperature (°C)		WSRTP
	CK	SM			CK	SM	
Jun-2010	21.86±0.41	20.59±0.34	< 0.001	May-2011	20.33±0.32	18.55±0.24	< 0.001
Jul-2010	25.48±0.23	24.18±0.20	< 0.001	Jun-2011	23.95±0.24	22.02±0.24	< 0.001
Aug-2010	25.37±0.48	24.30±0.42	< 0.001	Jul-2011	26.32±0.19	24.40±0.16	< 0.001
Sep-2010	21.45±0.45	20.85±0.41	< 0.001	Aug-2011	25.10±0.37	23.82±0.30	< 0.001
Oct-2010	15.85±0.46	16.17±0.33	0.090	Sep-2011	20.08±0.47	19.04±0.41	< 0.001
Nov-2010	11.28±0.31	11.54±0.26	0.029	Oct-2011	16.32±0.33	15.42±0.31	< 0.001
Dec-2010	6.33±0.43	7.48±0.32	< 0.001	Nov-2011	12.75±0.30	11.82±0.25	< 0.001
Jan-2011	2.10±0.13	3.18±0.11	< 0.001	Dec-2011	5.80±0.24	6.69±0.23	< 0.001
Feb-2011	5.00±0.30	5.32±0.27	0.012	Jan-2012	3.47±0.16	4.27±0.16	< 0.001
Mar-2011	8.61±0.45	8.20±0.33	0.016	Feb-2012	4.41±0.20	5.14±0.21	< 0.001
Apr-2011	15.54±0.68	13.99±0.50	< 0.001	Mar-2012	8.05±0.57	6.37±0.52	< 0.001

Values are given as means ± standard error of means. WSRT: Wilcoxon Signed Rank Test, a P value of <0.05 was considered statistically significant.

**Figure 8:**
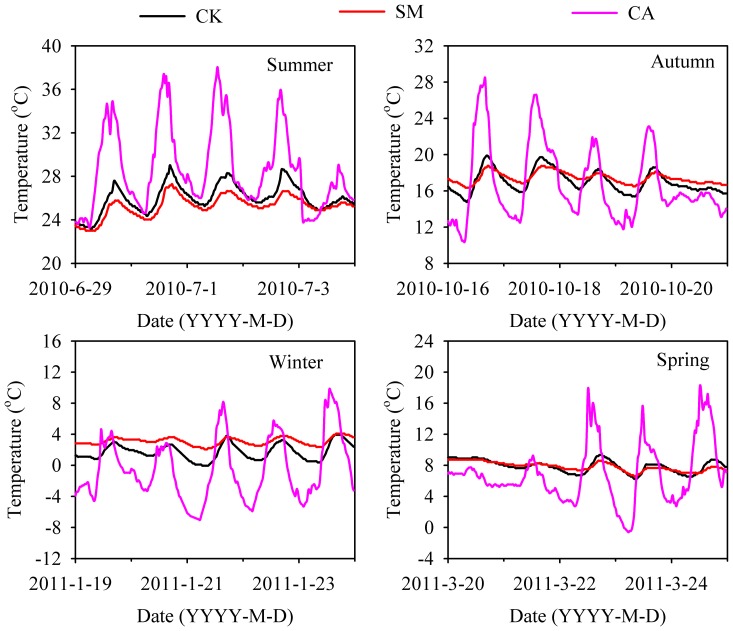
Typical diurnal air temperature trends for Summer, Autumn, Winter and Spring seasons within the canopy (CA) and soil temperatures under control (CK) and straw mulching (SM) treatment plots.

Four sets of typical diurnal trends (for summer, autumn, winter and spring) of CA and soil temperatures under both SM and CK conditions are presented in Fig. 8. In spring and autumn, when diurnal temperature range was large, soil temperature under mulch was lower during daytime, but higher at night. As daily radiation increased in summer, soil temperature under SM was always lower than that without mulch (CK). However, soil temperature was always higher in SM than in CK plots when the air temperature reached its minimum value in winter.

### Fruit yield

Differences in citrus fruit yields were recorded in the SM and CK plots in different years (Table 2). There was no significant difference in fruit yield and fruit quality between CK and SM treatments in 2010, while in 2011, this difference in fruit yield was significant. Interestingly, the citrus yield was completely destroyed (zero fruit yield) in the CK treatment plot in 2011 due to extremely low temperatures during the citrus overwintering stage. According to visual observation, almost 50 to 60% of the leaves of citrus trees died or etiolated as a result of a cold and dry weather conditions that prevailed. In SM plots, despite the large reduction in fruit production, some fruit yield was recorded. The citrus fruit yield was 10.3±9.3 t hm^−2^ for SM in 2011.

**Table 2:** Yield and fruit quality under the control (CK) and straw mulching (SM) treatments in 2010 and 2011.

Treatment	Yield (t hm^−2^)		Single fruit weight (g)		Equatorial diameter (mm)		Juice content (%)	
	2010	2011	2010	2011	2010	2011	2010	2011
CK	39.5±6.7 a	- -	172±32 a	- -	72.3±12.5 a	- -	42.5±4.0 a	- -
SM	43.2±5.6 a	10.3±9.3	168±7 a	179±42	70.8±3.8 a	76.0±10.9	41.2±5.1 a	40.9±3.6

Values are given as means ± standard error of means (n = 3). Values followed by different letters within a column are significantly different (P<0.05). Please note that the citrus crop was completely destroyed (zero citrus fruit yield) in the CK treatment plot in 2011 due to extremely low temperatures during the citrus overwintering stage.

## Discussion

Field observations indicated that the SM treatment significantly decreased the annual runoff as compared to the CK treatment. For example, Adekalu, et al. [13] found that elephant grass (*Pennisetum purpureum*) can be a good alternative for rice straw to effectively reduce runoff and increase infiltration on sloping lands in southwestern Nigeria. Lal [26] found mulching tilled soil with 4–6 t hm^−2^ of rice straw to be effective in reducing soil loss and runoff on slopes ranging from 1% to 15%. Earlier studies have extensively discussed how soil surface mulching reduces the runoff by buffering the ground from raindrop action [21], [27] and by modifying soil physical properties through the addition of litter and organic soil matter [28]. Furthermore, the absorption capability of straw provides additional pathways for water infiltration [21]. This trapped water in the straw is gradually released over several days, resulting in decreasing the velocity of surface flow and increasing infiltration. In general, surface runoff decreases with an increase of SM [13]. During the rainy season, a straw cover of 50% is necessary to significantly reduce runoff [29]. Results from the present study also provide indirect support for this conclusion, since the straw cover reached 100% in the SM plots, which was effective in controlling runoff water loss.

Reduced runoff means an improvement in the soil water status in the root zone and a reduction in soil loss, which in turn leads to reduced land degradation and crop water stress [30]. Moreover, due to the evaporation reducing property of the surface-placed straw layer, mulching (SM treatment) increased soil water storage on an average by 10 mm as compared to the CK treatment (Fig 5). The effect was particularly pronounced during dry periods, when no rain occurred. Similar results have also been reported earlier [13]. Adekalu, et al. [13] stated that the large pores of crop residues permit rapid infiltration of water into the soil but retard evaporation. Water moves back to the atmosphere across the straw pores almost entirely in the vapor phase. The straw tends to act as a one-way water valve for the soil, thus water remains in the soil longer and benefits growing plants. Consequently, more soil water content in the SM treatment plot mainly resulted from higher infiltration, lesser runoff, and lower evaporation than in the CK treatment plot.

Under SM, soil temperature has been reported to have increased [31] as well as decreased [18]. And this can be mainly attributed to differences in climatic conditions. This effect can be explained with two basic mechanisms as observed in our field experiment. In the SM treatment, the mulch layer reduced soil radiation absorption during daytime, while at nighttime it reduced the outgoing heat radiation from the soil. Moreover, the mulch layer contains a significant amount of pore space. The majority of this pore space is likely to be filled with air, and air is known to be a very good insulator. The air space in the mulched layer prevents energy conduction. Therefore, in our study the SM treatment had lower thermal conductivity than the non-mulch control (CK), and acted as an insulator during the warmer period and helped to retain soil heat during the colder period, resulting in smaller fluctuations in soil temperatures (Fig. 7 and 8). Chen, et al. [12] and Olasantan [15] also observed similar results.

Previous research has shown that SM is an effective method to improve crop yield and soil water utilization [32]. Li, et al. [32] found that SM increased soil water content and maize (*Zea mays L.*) yields. Kar and Kumar [33] reported that potato tuber yield was higher in the SM treatments than with bare flat planting in eastern India. However, application of SM is restricted in some places because it is liable to lower the soil surface temperature and lead to a reduction in yield [34], [35]. As pointed out by Doring, et al. [34], higher yields under mulch have mostly been attributed to increase soil water under arid and semiarid conditions [17], [35], while reduced yields under SM have also been reported and have been attributed to below-optimum soil temperature, reduced soil nitrate levels, and mulching too early [35]. In our study, the results indicate that the effect of SM on citrus fruit yield can be positive in extreme weather conditions. This was possibly because SM reduced the outgoing heat radiation from the soil and, thus, increased the soil temperature compared to the no mulching or CK treatment. Higher soil water content during the dry season in the SM treatment may also have attributed to some fruit yield vis-à-vis the complete fruiting failure in the CK treatment.

## Conclusions

From the comparison of runoff, soil water and temperature under both SM and CK conditions we conclude: (1) the surface runoff from the sloping citrus orchards were lower when the soil was mulching with straw than when it was unmulching. (2) Soil water storage in the top 100 cm of the soil profile was increased in the SM as compared to the CK treatment. However, this result was significant only in the dry season (Jan to Mar), and not in the wet season (Jul to Sep) for both years. (3) The mulch probably acted as an insulator, resulting in smaller fluctuations in soil temperature in the SM than in the CK treatment. The results of our study suggested that the small effects on soil water and temperature changes created by surface mulch had limited impact on citrus fruit yield in a normal year (e.g., in 2010). However, SM practices can positively impact citrus fruit yield in extreme weather conditions.

## Supporting Information

Table S1 **Soil bulk density at start of the field experiment in 2009.**
(XLS)

Table S2 **Field data and summary statistics of canopy air temperature for 2010 and 2011 at Xiaofuling experimental station.**
(XLS)

Table S3 **Field data and summary statistics of rainfall for 2010 and 2011 at Xiaofuling experimental station.**
(XLS)

Table S4 **Field data and summary statistics of runoff under control (CK) and straw mulching (SM) treatments during 2010 and 2011.**
(XLS)

Table S5 **Field data and summary statistics of soil water storage in the 0–100 cm soil profile under control (CK) and straw mulching (SM) treatments during 2010 and 2011.**
(XLS)

Table S6 **Field data and summary statistics of soil temperature under control (CK) and straw mulching (SM) treatment plots from May 2010 to March 2012.**
(XLS)
